# The Influence of an Orthopedic, Manual Therapy Residency Program on Improved Knowledge, Psychomotor Skills, and Clinical Reasoning in Nairobi, Kenya

**DOI:** 10.3389/fpubh.2017.00055

**Published:** 2017-03-20

**Authors:** Shala Cunningham, Joni McFelea

**Affiliations:** ^1^Department of Physical Therapy, Radford University, Roanoke, VA, USA; ^2^Department of Physical Therapy, University of Evansville, Evansville, IN, USA

**Keywords:** global health, physical therapy, residency program, manual therapy, Kenya

## Abstract

**Introduction:**

The purpose of this study was to describe the influence of a post-graduate orthopedic manual therapy residency program in Kenya on the development of physical therapists’ (PTs) knowledge and clinical reasoning related to the performance of a musculoskeletal examination and evaluation as compared to an experience-matched control group of PTs waiting to enter the program.

**Methods:**

A cross-sectional design was utilized in which 12 graduating residents and 10 PTs entering the residency program completed a live-patient practical examination to assess the knowledge, clinical reasoning, and psychomotor skills related to the examination and evaluation of musculoskeletal conditions. The assessment utilized was based on the tasks, procedures, and knowledge areas identified as important to advanced clinicians in the US as outlined by the Orthopaedic Description of Specialty Practice. Inclusion criteria included participation in or acceptance to the residency program, practice as a PT between 3 and 25 years, and 50% of workday being involved in direct patient care. Overall pass rates were analyzed using the Pearson chi-square and Fisher’s exact tests to determine if the graduating residents achieved significantly higher scores than experience-matched controls consisting of PTs entering the residency program.

**Results:**

PTs completing a post-graduate orthopedic manual therapy residency in Nairobi, Kenya, achieved higher scores and passing rates compared to their colleagues who had not completed a residency program as determined by a live-patient practical examination. Graduating residents demonstrated statistically significant higher scores in the categories of examination, evaluation, and diagnosis. The average live-patient practical examination score for PTs without residency training was 38.2%, and their pass rate was 0.0%. The average live-patient practical examination score for residency-trained PTs was 83.4%, and their pass rate was 92.3%. These findings are statistically significant (*p* < 0.001).

**Discussion:**

The study results suggest that the residency program had a positive influence on the residents’ ability to perform musculoskeletal examination and evaluation and to determine a treatment diagnosis. Future studies should be performed to determine if the improvements in assessment have a positive impact on clinical practice.

## Introduction

Kenya is a developing nation in eastern Africa with approximately 600 registered physical therapists (PTs) delivering care to a population of 44 million people (1). PTs in Kenya currently have the opportunity to earn a 3-year diploma or a Bachelor of Science degree in the field of physical therapy (2). According to the World Confederation for Physical Therapy, education for entry-level therapists should include 4 years of university-level courses. In addition, PTs should be committed to pursuing educational opportunities following entry-level education to promote the development of the profession (3). Access to advanced instruction, fundamental to promoting educational development, is limited throughout the country of Kenya. One restricting factor has been the shortage of PTs with advanced degrees and specialty training to offer educational opportunities (4). To assist with promotion of skill advancement, clinical reasoning, and use of current evidence in practice, the Kenya Medical Training College Higher Diploma Program offered the first post-graduate residency program, which was administered by the Jackson Clinics, in 2012. The degree of Higher Diploma corresponds to the current educational structure of the region and denotes advanced training beyond the entry-level PT. The program is focused on a long-term commitment to the region and initiated a train-the-trainer program to transit the administration of the residency program to local administrators.

Similar to the medical model, a clinical residency program is a structured experience for PTs following entry-level education that is designed to advance the therapist’s knowledge, skills, clinical reasoning, and attributes in a specific area of practice (5). The residency experience combines opportunities for ongoing mentoring to the resident, including required written and practical examinations, with a foundation of evidence-based practice and course work designed to provide a theoretical basis for advanced practice (5). Several key activities have been noted for promoting the success of continuing education in effecting clinical practice and improving patient outcomes (6). Many of these activities have been incorporated into the residency program: a needs assessment, interactive education, multiple methods of instruction, multiple exposures to the material, and case-based learning. In addition, the program emphasizes and provides clinical mentoring within the resident’s current place of employment. This allows for the incorporation of deliberate practice into learning activities and feedback for performance correction (7). In addition, active reflection on the outcome of the treatment, as compared to previous experience, is encouraged to facilitate the transfer of knowledge from the program to clinical practice.

Current published literature suggests that graduate residents subjectively value residency education for its influence on clinical reasoning and professional development. Utilizing survey methodology, graduates of a residency program in California reported a positive impact of the program on the ability to perform a comprehensive evaluation, utilize clinical reasoning in treatment decisions, and implement an effective treatment plan employing scientific literature (8). In addition, graduates reported career advancement through promotions and increases in salary (8). A second study expanded on the survey by introducing a comparison group. It demonstrated that residency graduates are more likely to become board certified physical therapy specialists and to participate in providing educational instruction than non-residency graduates (9). Likewise, the residency graduates earn a higher income when compared to non-residency-trained PTs with similar experience (9).

Despite the positive subjective reports of improvement in knowledge and clinical reasoning, residency programs in the US have not been shown to improve patient outcomes (8). Utilizing the Focus on Therapeutic Outcomes (FOTO) database, patient outcomes have been compared between PTs without residency or fellowship education to PTs who have completed an orthopedic residency program accredited by the American Board of Physical Therapy Residency and Fellowship Education. There was no difference in functional outcomes between the two groups. Furthermore, the non-residency-trained PTs achieved functional outcomes in fewer treatment visits (10).

The study was limited, however, by the use of retrospective data collected from commercial databases. These drawbacks were outlined by Resnik and Hart when they attempted to utilize the FOTO database to identify expert clinicians (11). There is no process, when one accesses such a database, to determine how patients were selected. If a patient did not complete an episode of care due to failure to improve or worsening of the condition, their information would not be captured by the database as a discharge outcome would not be completed. Patients with poor outcomes may not have been encouraged to complete the discharge outcome tool. These scenarios may have led to bias in the clinical database. Furthermore, there was no ability to adjust the data analysis for covariates, such as comorbid conditions and complexity of the patient presentation. Residency-trained therapists have reported an increased comfort level in assessing and treating complex patients, which may result in a patient population with difficult clinical presentations and, therefore, poorer prognosis. Classification of patients to improve homogeneity may provide additional insight into outcomes. Last, the outcome measures may not reflect factors considered important to both the patient and therapist. These factors make classifying therapists as expert versus average in assessment and treatment skills difficult with the utilization outcomes measures alone.

In all health-care professions, the development and progression of clinical reasoning skills is perceived as a key factor in distinguishing expert from novice clinicians (12). It has been shown that clinical reasoning has three distinct areas of development. These areas include declarative knowledge or content specific knowledge, practical knowledge requiring multiple approaches for an identifiable clinical problem, and reflective practice (13). Due to its complex nature, clinical reasoning is difficult to objectively assess. Three areas of clinical reasoning are reportedly observable and measureable. These areas include collection of important of key information, diagnosis outcomes, and how clinicians integrate new patient information into their existing knowledge organization to gain an improved understanding of the clinical problem (13–15).

Insufficient research is available to evaluate the effectiveness of residency programs to progress the development of clinical reasoning. Clinical reasoning development has been associated with declarative knowledge, practical knowledge, and reflective practice (14, 15). Each of these distinct areas is addressed in the current post-graduate residency education model utilized in the US through the instruction of content-specific didactic information, clinical mentoring, and self-assessment (5). In addition, several key aspects of effective continuing medical education have been incorporated into residency programs. The accepted theory that residency training assists with the development of clinical reasoning associated with expert practice has not been established in the literature. An attempt to measure this construct may provide additional insight into the ability of residency training to improve patient care provided. The purpose of this study was to determine the influence of a post-graduate, orthopedic residency program on the participant’s knowledge, clinical reasoning, and psychomotor skills related to the examination and evaluation of musculoskeletal conditions.

## Materials and Methods

A cross-sectional design was utilized in which 12 graduating residents and 10 experienced matched PTs entering the residency program completed a live-patient practical examination to assess their knowledge, clinical reasoning, and psychomotor skills related to the examination and evaluation of patients with musculoskeletal conditions. The examinations were performed over a 5-day period in Nairobi, Kenya, at the Kenya Medical Training College. The assessment tool utilized was based on the American Board of Physical Therapy Specialties Dimensions of Specialty Practice in Orthopedics. The assessment tool was comprised of 58 items within four categories of performance: examination, evaluation, diagnosis, and prognosis. Cronbach’s alpha was calculated to determine internal consistency for items in each of the categories and were 0.871 for examination, 0.818 for evaluation, 0.836 for diagnosis, and 0.603 for prognosis.

The study participants completed the live-patient practical examination with no information about their patient’s current condition, signs and symptoms, and reason for referral. The study participants were asked to complete the live-patient practical examination and evaluation and to determine a clinical musculoskeletal diagnosis. The assessment form that was utilized assessed the collection of key information and psychomotor skills. Following the examination and evaluation, interviews were performed with the participants to assess the mental processes associated with their clinical reasoning. This included how the PT integrated information into their existing knowledge framework to gain an improved understanding of the clinical problem. Participants were asked to describe the nature of the patient’s symptoms, the anatomical structures involved in the dysfunction, and probable causes of the patient’s condition. Furthermore, discussion occurred regarding how they prioritized functional limitations, interpreted data regarding the irritability of the condition, and identified psychosocial factors that may influence rehabilitation.

Two examiners who were well versed in the use of the assessment form performed the assessments. The examiners were blinded to the participants. Descriptive statistics, including frequency counts for each of the 58 items assessed during the examination, were calculated *via* IBM SPSS 22. In addition, overall pass rates were analyzed using the Pearson chi-square and Fisher’s exact tests to determine if the graduating residents achieved significantly higher scores than PTs entering the residency program.

## Results

Demographic information of the two groups is listed in Table 1. The graduating residents completed a significantly greater number of outpatient visits during an 8-h day than the entering residents (*F* = 9.041, with a significance level of 0.006). Graduating residents reported a positive shift in patient care load toward more outpatient care as they developed new skills through the residency program.

**Table 1:** **Participant demographic information**.

	Residents	Mean	SD	SEM	Levene’s test	
					*F*	Sig
Age	Entering	33.30	9.627	3.044	1.607	0.219
	Graduating	35.54	5.811	1.612		
Years practicing as a physical therapist	Entering	9.40	8.708	1.754	0.230	0.637
	Graduating	11.08	6.589	1.827		
Percent time in patient care	Entering	75.50	12.994	7.904	1.036	0.320
	Graduating	80.77	15.525	4.306		
Number of outpatient visits in an 8-h day	Entering	5.60	3.098	0.980	9.401	0.006
	Graduating	8.38	5.839	1.619		

The live-patient examination assessment results were analyzed for significant differences between the two groups. The assessment category of examination focused on the ability to collect key information regarding the patient in order to make deductions regarding the patient’s diagnosis and prognosis. Behaviors listed under evaluation, diagnosis, and prognosis best describe how clinicians integrate patient information into their existing knowledge organization to gain an improved understanding of the clinical problem. In addition to considering each individual behavior, the categories of examination, evaluation, diagnosis, and prognosis were assessed for significant differences between the graduating and incoming residents using the chi-square analysis and Fisher’s exact test. Table 2 includes a summary of the category analysis. There was no significant difference in the category of prognosis between the cohorts.

**Table 2:** **Live-patient examination category assessment**.

Behavior category	Graduating satisfactory performance	Entering satisfactory performance	Chi square	Fisher’s exact two sided
Examination	277	162	69.811	<0.001
Evaluation	160	86	59.262	<0.001
Diagnosis	20	7	12.564	<0.001
Prognosis	17	15	7.628	–

The average live-patient practical examination score for PTs without residency training was 38.2%, and their pass rate was 0.0%. The average live-patient practical examination score for residency-trained PTs was 83.4%, and their pass rate was 92.3%. These findings are statistically significant (*p* < 0.001). Utilizing a live-patient examination allowed for the assessment of performance in addition to competence (knowledge). These results demonstrate the effectiveness of the training on knowledge, skill development, and most importantly, the clinical reasoning associated with expert practice.

## Discussion

This study represents a novel attempt to objectively measure clinical reasoning of PTs with residency education. PTs completing a post-graduate orthopedic manual therapy residency in Nairobi, Kenya, achieved higher scores and passing rates compared to their colleagues who had not completed a residency program as determined by their performance of a live-patient practical examination. In three of the four assessment categories, the residents demonstrated statistically significant higher scores compared to the experienced matched controls. The residency graduates were able to more effectively collect necessary key information and collectively interpret this information to determine a definitive diagnosis. There was no difference between the two groups with regard to prognosis. The ability of the residents to determine a prognosis was measured by their (1) proficiency in choosing reassessment measures to determine initial and long-term responses to intervention and (2) prioritization of interventions based on the impairments noted during their examination. Assessment of this category was done through an interview with the resident and with limited cues to the participant. The assessment tool has been revised to provide additional cues to assist the participant in understanding the information being requested.

Results from this pilot study suggest the residency program was successful in promoting clinical reasoning. These results are similar to the documented subjective perceptions of residency graduates in the US. In addition, residency graduates noted a shift in their clinical practice toward an orthopedic population as their skills improved through participation in the residency. This change in clinical practice may reflect the perception of these PTs as experts in the field.

The residency program incorporated many of the aspects of continuing education associated with improved performance and clinical outcomes in medicine. The utilization of an authentic patient assessment, through the live-patient practical examination, provided a measure of not only competence but also performance. Although the theoretical link between expert practice and patient outcomes has not been substantiated, the residency program was successful in promoting clinical reasoning associated with expert practice. This program may provide the framework for the development of additional residency programs in countries with limited educational resources. The development of residency programs that can influence the ability of PTs to provide treatment efficiently and effectively may ultimately assist in serving community physical therapy needs. According to the United Nations, 80% of all disabled persons live in rural areas within developing countries without access to adequate medical treatment (16). PTs in these countries have an important role in the treatment of disability and maintenance of quality of life.

Limited medical documentation in Kenya prohibited a prospective study using outcome measures to quantify changes in treatment results and clinical practice. Future studies should be performed to determine if the improvements in clinical reasoning have a positive impact on clinical practice.

## Ethics Statement

The Kenya Medical Training College Ethics and Research Committee and the University of Evansville Institutional Review Board approved this research. Current residents and entering residents were informed about the upcoming study by the residency program coordinator in August 2014. In September 2014, the primary investigator traveled to Kenya to visit each cohort and discuss the purpose of the study, procedures associated with the study (access to practical examination results), and requirements for time involvement. Informed consent was explained and the investigator was present for questions regarding the project. The residents’ examiners and instructors did not have access to a list of residents consenting for the study. All residents completed the practical examination as a required by the residency program. Additional time to complete demographic information was approximately 5 min.

## Author Contributions

SC developed the research methodology and performed the data analysis for the study. JM assisted with the methodology development and manuscript.

## Conflict of Interest Statement

The authors declare that the research was conducted in the absence of any commercial or financial relationships that could be construed as a potential conflict of interest.
